# Genotype-Associated Differential NKG2D Expression on CD56+CD3+ Lymphocytes Predicts Response to Pegylated-Interferon/ Ribavirin Therapy in Chronic Hepatitis C

**DOI:** 10.1371/journal.pone.0125664

**Published:** 2015-05-12

**Authors:** Po-sung Chu, Hirotoshi Ebinuma, Nobuhiro Nakamoto, Kazuo Sugiyama, Shingo Usui, Yuko Wakayama, Nobuhito Taniki, Akihiro Yamaguchi, Shunsuke Shiba, Yoshiyuki Yamagishi, Takaji Wakita, Toshifumi Hibi, Hidetsugu Saito, Takanori Kanai

**Affiliations:** 1 Department of Gastroenterology and Hepatology, Division of Internal Medicine, School of Medicine, Keio University, Tokyo, Japan; 2 Department of Virology II, National Institute of Infectious Diseases, Tokyo, Japan; 3 Center for Advanced IBD Research and Treatment, Kitasato Institute Hospital, Kitasato University, Tokyo, Japan; 4 Division of Pharmacotherapeutics, School of Pharmacy, Keio University, Tokyo, Japan; Karolinska Institutet, SWEDEN

## Abstract

Hepatitis C virus (HCV) genotype 1 infections are significantly more difficult to eradicate with PEG-IFN/ribavirin therapy, compared to HCV genotype 2. The aim of this work is to investigate the difference of immunological impairments underlying this phenomenon. Pre-treatment NKG2D expression on peripheral CD56+CD3+ lymphocytes and CD56+CD3− NK cells from cases of chronic hepatitis C were analyzed and assessed by treatment effect. Two strains of HCV were used to co-incubate with immune cells in vitro. NKG2D expression on peripheral CD56+CD3+ lymphocytes, but not NK cells, was significantly impaired in genotype 1 infection, compared to genotype 2. When peripheral blood mononuclear cells from healthy donors were co-incubated with TNS2J1, a genotype 1b/2a chimera strain, or with JFH1, a genotype 2a strain, genotype-specific decrease of NKG2D on CD56+CD3+ lymphocytes, but not NK cells, was observed.　Pre-treatment NKG2D expression on peripheral CD56+CD3+ lymphocytes significantly correlated with reduction in serum HCV RNA levels from week 0 to week 4, and predicted treatment response. Ex vivo stimulation of peripheral CD56+CD3+ lymphocytes showed NKG2D expression-correlated IFN-γ production. In conclusion, Decreased NKG2D expression on CD56+CD3+ lymphocytes in chronic HCV genotype 1 infection predicts inferior treatment response to PEG-IFN/ribavirin therapy compared to genotype 2.

## Introduction

More than 70% of acute hepatitis C virus (HCV) infections become persistent and lead to chronic hepatitis [1, 2]. Longer persistency of HCV infection increases the rate of progression to liver cirrhosis [3]. There are several immunological mechanisms that favor HCV persistency, including disruption of pattern-recognition receptor function, impaired innate interferon function, increase of regulatory T cells, and exhaustion of T cells [2, 4–6]. For immune cells with cytotoxicity, including natural killer (NK) cells, natural killer T (NKT) cells, or CD8+ cytotoxic T lymphocytes (CTLs), one of the important mechanisms for the eradication of HCV-infected hepatocytes is through interferon (IFN)-γ, alongside many of the immunoregulatory functions of this cytokine [2, 4]. IFN-γ secretion can be induced by cross-linking natural killer group 2, member D (NKG2D), an activating receptor for MHC class I chain-related protein A/B (MICA/B) or other ligands [7–9]. NKG2D is previously known as an NK cell marker, however, other cytotoxic cells, including CTLs, NKT cells, or γδT cells, also express NKG2D [7, 9–13].

Human CD56+CD3+ lymphocytes are T cells expressing both T cell and NK cell markers [10]. Classical invariant NKT (iNKT) cells, which are CD1d-restricted with a very limited repertoire of T cell receptors, are more sophisticatedly studied in mice than in human [10]. Although not all CD56+CD3+ lymphocytes are classical iNKTs, by the original definition, they belong to the broader group of NKT cells. Some researchers refer human CD56+CD3+ lymphocytes as natural T (NT) cells [10, 14–16]. Because human iNKT cells are relatively sparse in frequency (about 1–2% in peripheral blood and less than 1% in livers)[17, 18], and multiple isoforms of human CD1 exist, iNKT cells are less likely to be the only effective NKT cells in human [19]. Human livers, as primary targets for HCV infection, are enriched of CD56+CD3+ lymphocytes making up about 30% of hepatic CD3+ cells [10, 15, 16]. Unlike classical T cells, CD56+CD3+ lymphocytes may produce large quantities of both Th1 and Th2 cytokines, and alongside human iNKT cells, are considered important innate immune cells as first-line defense against pathogens, including viruses [14]. Although reduced NKG2D expression on “NK cells” in acute [20] and chronic [21] HCV infections have been reported, the NKG2D expression on “CD56+CD3+ lymphocytes” and its immunological role in chronic HCV infection has not yet been fully studied.

Over the past two decades, interferon-based antiviral therapy has been the standard treatment for chronic hepatitis C. Multiple viral and host factors can affect the goal of reaching sustained virologic response (SVR). The most prominent viral factor is the genotype of HCV[2]. Genotype 1 infection is significantly more difficult to eradicate by pegylated-interferon (PEG-IFN)/ ribavirin therapy than genotype 2 [2, 22–24]. The underlying immunological differences aroused by different HCV genotypes must exist, however, they are not yet fully elucidated.

In this study, followed by the evaluation of the expression of NKG2D on peripheral blood CD56+CD3+ lymphocytes, CD56+CD3- NK cells and CD3+CD56−CD8+ CTLs between chronic HCV infections of genotype 1 and genotype 2, we revealed that NKG2D expression on CD56+CD3+ lymphocytes predicts treatment response, and may be key to the immune mechanism mediating genotype difference in chronic HCV infections.

## Materials and Methods

### Study Subjects

According to the ethical guidelines of the 1975 Declaration of Helsinki, the institutional review board of Keio University School of Medicine approved the study protocols specifically before we started this study. Recruited study subjects provided prior written informed consents, which included blood sampling and study participation. Study protocols were as follows:

- For the evaluation of NKG2D expression, sixty-six patients with chronic HCV infection, including fifty-three with genotype 1, and thirteen with genotype 2, were included. No patient with hepatocellular carcinoma was included. NKG2D expression on peripheral blood mononuclear cells (PBMCs) was analyzed before treatment. Detailed definition of flow cytometry analyses and gating strategies are explained below. Clinical characteristics are summarized in Table 1, and the detailed statistics were included in supporting S1 Table. PBMC analyses from nine healthy volunteer donors were used as controls.
- Thirty patients with chronic HCV infection, including seventeen with genotype 1 and thirteen patients with genotype 2, were analyzed for treatment response. PEG-IFNα-2b (Schering-Plough KK, Tokyo, Japan) and ribavirin (Schering-Plough KK) were administered in combination at doses according to the manufacturer’s instructions. Treatment duration was determined by the treatment guideline from the Japan Ministry of Health, Labour and Welfare [25]. Patient characteristics are shown in supporting S2, S3, and S4 Tables. Undetectable plasma HCV RNA (< 15IU/ml) at week 4 is defined as rapid viral response (RVR). Negative plasma HCV RNA at 24 weeks after the end of treatment is considered to have a sustained virological response (SVR). Patient whose HCV RNA declines more than 2 log10 at week 12 but is positive at week 24 is defined as partial responder (PR). Patient whose HCV RNA declines less than 2 log10 at week 12 is defined as null responder (NR).
- Fourteen patients with chronic HCV genotype 1 infection were included for the evaluation of NGK2D expression on IFN-γ production. Clinical characteristics of these patients are summarized in supporting S5 Table.

**Table 1:** Clinical characteristics of cases of genotype 1 and genotype 2.

Characteristics	Units	All cases	Genotype 1	Genotype 2	*p*
HCV genotype, 1: 2	-	53:13	-	-	-
Patient number, n		66	53	12	-
Age	Years	Median 62	Median 62	Median 60	0.25^a^
		(51–67)	(52–68)	(46.5–65)	
Gender, M:F	-	35:31	30:23	5:8	0.33^b^
Liver histology,	-	11/9/5/5 (30)	7/5/4/4 (20)	3/4/1/1 (10)	0.71^b^
F1/F2/F3/F4					
(data available)					
HCV-RNA	Log	Median 6.6	Median 6.7	Median 6.5	0.34^a^
	IU/ml	(6.1–7.0)	(6.0–7.0)	(6.25–6.65)	
PLT count	x1000/μl	170±54	166±53	185±56	0.23^c^
Serum albumin	g/dl	4.0±0.4	4.0±0.5	4.0±0.3	0.93^c^
Total	mg/dl	176±36	177±38	169±28	0.47^c^
Cholesterol					
ALT	IU/L	65±61	60±57	80±74	0.28^c^
ALP	IU/L	260±116	249±111	301±133	0.15^c^
WBC	/μl	4720±1450	4750±1470	4580±1430	0.71^c^
NKG2D	%	Median 75.8%	Median 72.3%	Median 90.1%	<0.0001^a^
expression on		(64.3%-83.4%)	(62.7%-82.2%)	(51.9%-81.4%)	
CD56+CD3+					
NKG2D	%	Median 68.9%	Median 66.5%	Median 77.0%	0.39^a^
expression on		(50.2%-81.1%)	(49.6%-82.0%)	(51.9%-81.4%)	
CD56+CD3-					
NKG2D	%	Median 87.4%	Median 84.3%	Median 92.9%	0.0048^a^
expression on		(77.1%-93.8%)	(74.1%-93.2%)	(90.4%–95.7%)	
CD8+ T cells					

^a^ Statistics are analyzed by Mann-Whitney U-test. IQRs are shown in the parentheses.

^b^ Statistics are analyzed by Student’s t-test. Data are shown as mean± standard deviation.

^c^ Statistics are analyzed by Fisher’s exact test.

Abbreviations: ALT, alanine aminotransferase, ALP, alkaline phosphatase.

### Flow Cytometry Analysis

**Fig 1:**
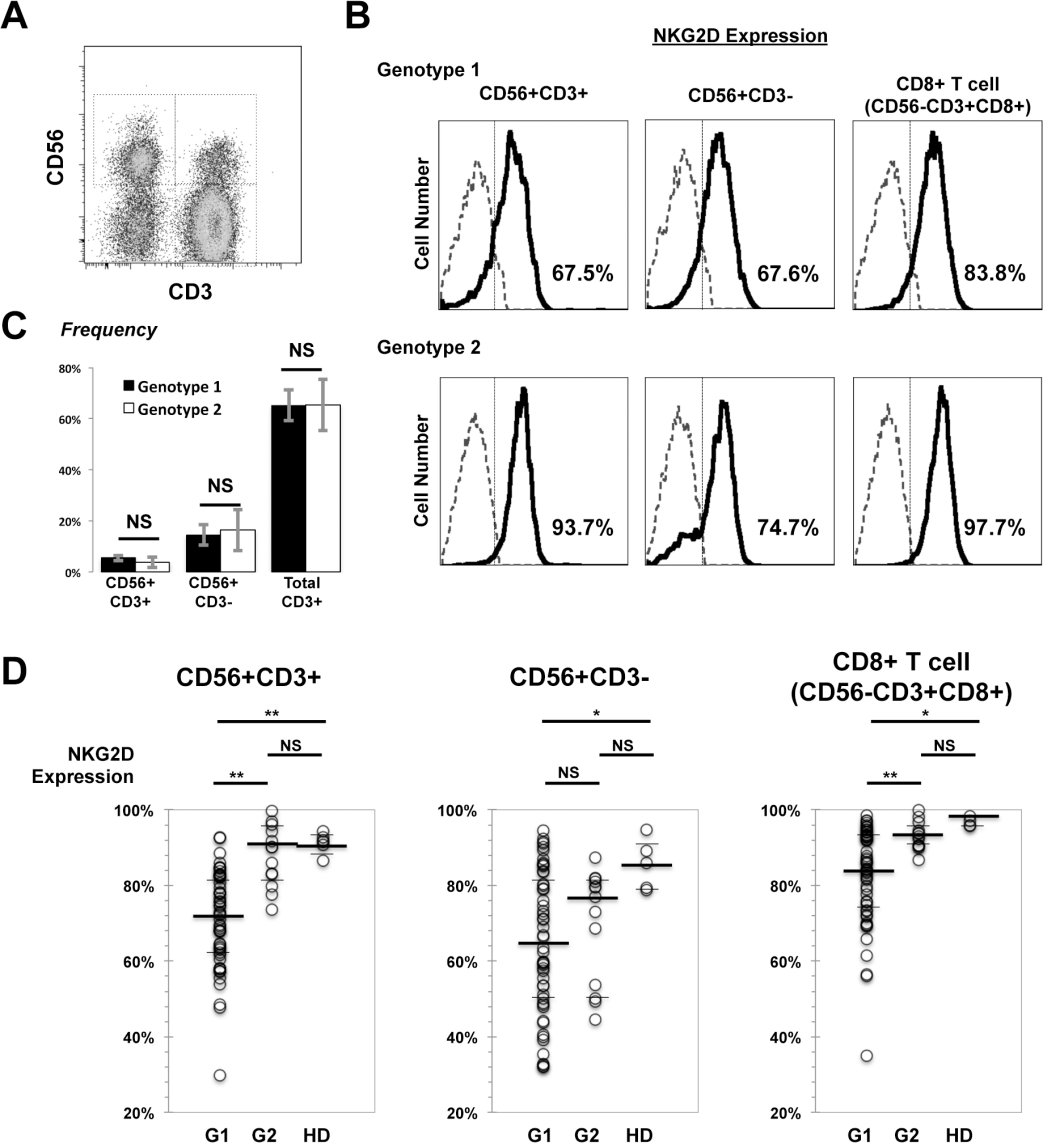
NKG2D expression on CD56+CD3+ lymphocytes in HCV genotype 1 infection is significantly impaired. PBMCs were collected and analyzed by flow cytometry. (A) Representative flow cytometry plot shows CD56+CD3+ lymphocytes, NK (CD3-CD56+) cells and T lymphocytes (CD3+CD56-) gated on SSC_low_ compartment. (B) Histograms from representative genotype 1 HCV infected (upper) and genotype 2 (lower) show NKG2D expression on CD56+CD3+ lymphocytes, NK cells and CD8+ T cells. Isotype controls are shown as dashed lines. Percentages are for NKG2D positive levels in corresponding compartments. (C) Peripheral frequency (compared to total SSC_low_ cells) stratified by HCV genotypes. (D) NKG2D expression on CD56+CD3+ lymphocytes, NK cells and CD8+ CTLs was compared by HCV genotypes (G1, genotype 1, n = 53; G2, genotype 2, n = 13), with control data from healthy donors (HD, n = 9). Statistics were analyzed by Mann-Whitney U-test. Medians, IQRs were presented as bars. Please refer to Table 1 for exact values *P < 0.05, **P < 0.01, NS; not significant.

Eight to sixteen milliliters of whole blood was drawn and PBMCs were isolated with BD Vacutainer CPT (Becton Dickinson, Franklin Lake, NJ, USA), containing 1.0 ml of 0.1 M sodium citrate, 3 g of polyester gel, and 2.0 ml of polysaccharide sodium diatrizoate. After centrifugation at 1500 × g for 25 minutes, PBMCs on the gel phase were harvested, and washed twice with RPMI-1640 medium (Sigma Aldrich, St. Louis, MO, USA) supplemented with 10% bovine albumin. Isolated PBMCs were incubated with specific fluorescence-labeled monoclonal antibodies (mAbs) at 4°C for 30 min. Anti-human mAbs including CD3 (FITC, BD), CD56 (PE-Cy7, BD), CD4 (APC, BD), CD8 (APC-Cy7, BD), 7-amino-actinomycin D (7-AAD) and NKG2D (PE, clone 1D11, BioLegend, San Diego, CA, USA) were used. Dead cells were excluded from assessment by 7-AAD. Irrelevant anti-rat isotype antibodies (BD) were used to assess background fluorescence. Stained cells were analyzed using FACS Canto II (BD), and the data was analyzed using FlowJo software (Tree Star Inc., Ashland, OR, USA). Gating strategies are illustrated in supporting S1 Fig NKG2D expression is defined as the percentage of NKG2D-positive cells compared to isotype controls, as illustrated in Fig 1B.

### Intracellular IFN-γ Production

Isolated PBMCs were adjusted to 2 × 10^6^/ml, and treated with brefeldin A (Sigma Aldrich, final concentration of 10 μg/ml), and at the same time, phorbol 12-myristate 13-acetate (PMA, Sigma Aldrich, final concentration of 10 ng/ml), and ionomycin (Sigma Aldrich, final concentration of 1 μg/ml) were added in the stimulation group. After 4 hours of incubation at 37°C with 5% CO_2_, cells were harvested, washed, and then stained for surface antigens including CD3 (APC), CD56 (FITC), and 7-AAD. After permeabilization and fixation with cytofix/cytoperm solution (BD) for 15 minutes, cells were stained with mAbs against IFN-γ (PE, BD) for 30 min at 4°C, washed twice, and then fixed in 1% paraformaldehyde. The samples were analyzed by flow cytometry.

### HCV Genotyping

HCV genotypes were determined by enzyme immunoassay according to previously reported methods [26]. According to Simmonds classification [27], serotype 1 corresponded to genotypes 1a and 1b, whereas serotype 2 corresponded to genotypes 2a and 2b. In Japan, 1b (about 70%) and 2a (about 20%) are the majority genotypes [28].

### Viral Kinetics

Plasma HCV RNA load was measured using COBAS TaqMan HCV test, with a lower limit of detection of 15 IU/ml (Roche Diagnostics Co, Ltd, Tokyo, Japan).

### *IL28B* SNP Genotyping

A single nucleotide polymorphism (SNP) of *IL28B* was evaluated in patients with genotype 1 infection [29]. We selected *rs8099917*, which was previously reported as the predictive factor of SVR by IFN-based therapies in Japan [29, 30], to determine *IL28B* SNP. This SNP is located within the same haplotype block as *rs12979860*, reported by Ge et al. [31], and are associated with each other [30]. TT at *rs8099917* is advantageous and defined as major, whereas TG or GG is minor.

### Amino Acid Substitutions

The nucleotide sequences of ISDR in the nonstructural 5A region (NS5A, amino acids (aa) 2209–2248) and core protein (aa70 and aa91) were determined by direct sequencing of PCR amplified materials. Mutant-type ISDR was defined as containing two or more than two amino acid substitutions [32]. Substitutions at aa 70 of arginine (Arg70, wild type) to glutamine/histidine (Gln70/His70), and at aa 91 of leucine (Leu91, wild type) to methionine (Met91) were determined [33].

### Co-incubation of HCV and PBMCs

PBMCs from healthy volunteer donors were isolated. JFH1, an HCV genotype 2a strain [34], and TNS2J1, a chimeric HCV strain of the structural region from genotype 1b and the non-structural region from genotype 2a [35], were used to infect hepatoma cell line Huh7.5. After several passages, supernatants enriched of infectious viral particles were concentrated by Amicon Ultra-15 (100,000 NMWL membrane; Merck Millipore, Billerica, MA). Supernatants from naïve Huh7.5 cultures were used as controls. Viral titers were determined as fluorescent focus forming units (FFU) in Huh7.5 cells. PBMCs were in contact with concentrated HCVs with a multiplicity of infection (MOI) of 0.5 FFU/cell. NKG2D expression on immune cells was determined by flow cytometry before treatment and after 48-hours of co-incubation.

### Statistics

Data were analyzed using JMP9 (SAS Institute, Inc. Cary, NC) and are expressed as mean ± standard deviation (SD) for Student’s t-test, or median with IQRs shown for Mann-Whitney U-test as appropriate. Receiver operating characteristic (ROC) analysis was performed for confirming the usefulness of NKG2D expression on CD56+CD3+ lymphocytes to predict SVR after PEG-IFN/ ribavirin therapy. Categorical data were analyzed using Fisher’s exact test. Differences were considered statistically significant when P < 0.05.

## Results

### NKG2D Expression on CD56+CD3+ Lymphocytes in HCV Genotype 1 Infection is Significantly Impaired

**Fig 2:**
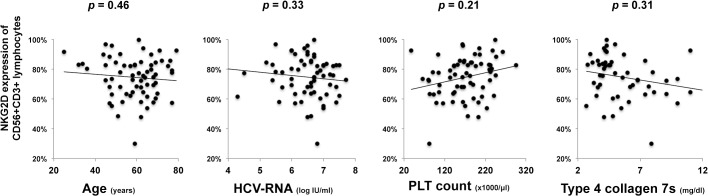
NKG2D expression on CD56+CD3+ lymphocytes has no significant correlation to age, HCV-RNA levels or liver fibrotic degree. PBMCs were collected and analyzed by flow cytometry for expression of NKG2D on CD56+CD3+ lymphocytes. Statistics of data including age, HCV-RNA, peripheral platelet count, and serum type IV collagen 7s levels were analyzed for correlation. Please refer to supporting S1 Table for detailed values.

PBMCs from patients of chronic HCV infection were isolated and analyzed for the surface expression of NKG2D by flow cytometry. The two groups of patients with HCV genotype 1 and 2 infection showed no statistical significant differences in age, gender, fibrotic stages or other characteristics (Table 1). NKG2D-expressing cells were gated as CD56+CD3− (NK cells), CD56+CD3+, and CD56−CD3+CD8+ (CTL) cells from the side-scatter low lymphocyte compartment, respectively (Fig 1). No significant differences in peripheral blood frequency of total T cells, NK cells, CD56+CD3+ lymphocytes were observed between genotype 1 and genotype 2 infections. NKG2D expression levels on CD56+CD3+ lymphocytes were significantly impaired in patients with HCV genotype 1 infection, compared to genotype 2 (Fig 1D, left). A similar tendency was observed with CD8+ T cells. However, between genotypes, no significant difference was observed in NK2GD expression on NK cells (Fig 1D). Furthermore, NKG2D expression on CD56+CD3+ lymphocytes did not correlate with age, HCV-RNA levels, or status of clinical liver fibrosis as assessed by blood platelet count and serum type 4 collagen 7s levels (Fig 2). Significantly different degrees of NKG2D expression induced by different HCV genotypes were noticed on CD56+CD3+ lymphocytes or on CD8+ T cells, but not on NK cells.

### Association of Differential NKG2D Expression on CD56+CD3+ Lymphocytes from Chronic Genotype 1 Infection and Other Known Predictive Factors

**Fig 3:**
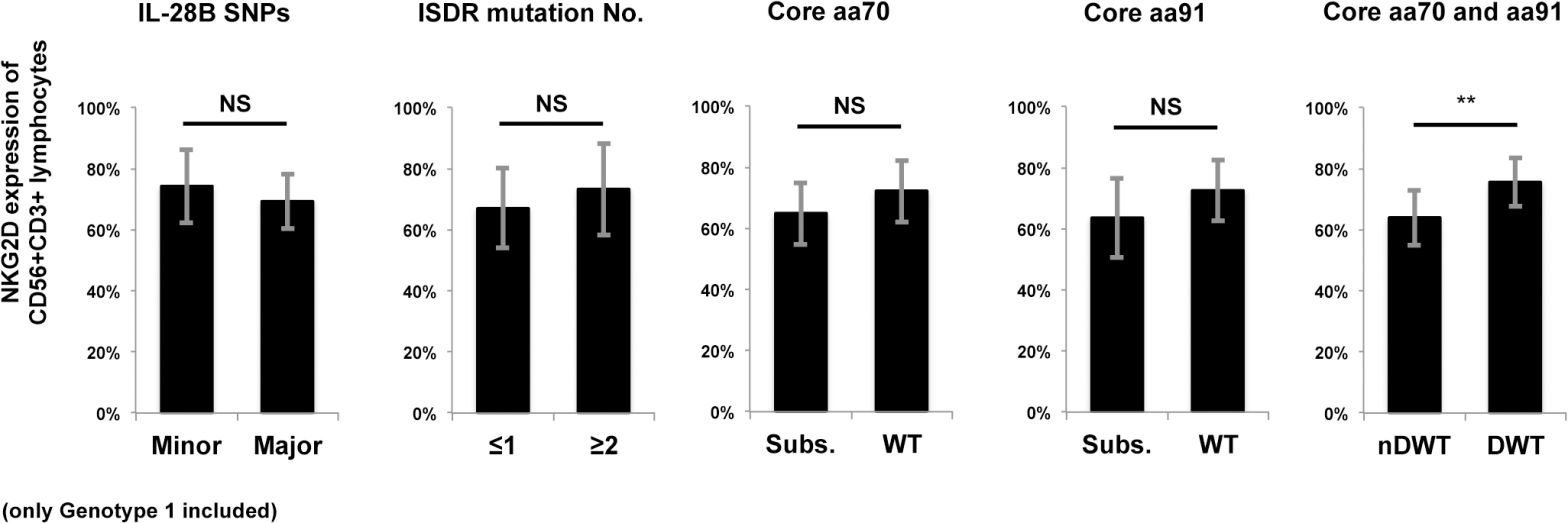
Differential NKG2D expression on CD56+CD3+ lymphocytes from chronic genotype 1 infection is associated with core protein aa substitutions. Cases of HCV genotype 1 infection were determined for the *IL28B* SNP polymorphism at *rs8099917*, mutation numbers of ISDR on NS5A (aa2209-aa2248) and core protein substitution at aa70 or aa91 before treatment. NKG2D expression on CD56+CD3+ lymphocytes was compared between *IL28B* genotype major (TT at *rs8099917*, n = 35) or minor (TG or GG, n = 18), ISDR mutation numbers ≤1 (n = 34) or ≥2 (= 19), aa70 substituted (subs, n = 17) or wild type (n = 36), aa91 substituted (n = 19) or wild type (n = 34), double wild type (DWT, n = 25) at aa70 and aa91 or non-double wild type (nDWT, n = 27). Data show mean ± SD. **P < 0.01. NS: not significant.

Since NKG2D expression on CD56+CD3+ lymphocytes differed between genotypes 1 and 2 (Fig 1C), and showed a differential range of expression in genotype 1, we hypothesized that NKG2D expression on CD56+CD3+ lymphocytes may correlate with treatment response. Thus, we attempted to identify whether any correlation with other known predictive factors exist, including IL28B polymorphism [30, 31], ISDR amino acid mutations [24, 32, 36], and core protein substitution at aa70 or aa91 [33]. Of fifty-three patients with genotype 1 infection included in this study, *IL28B* SNPs at *rs8099917*, mutation numbers of ISDR on NS5A (aa2209-aa2248) and core protein mutation at aa70 or aa91 were determined. In this cohort, NKG2D expression on CD56+CD3+ lymphocytes showed no difference when stratified by *IL28B* SNPs (major vs. minor) or ISDR mutation numbers (≤1 vs. ≥2). However, a significant difference when stratified by core protein determination at aa70 and aa91 (double wild-type or not, Fig 3) was noticed. This implies that differential NKG2D expression on CD56+CD3+ lymphocytes in chronic genotype 1 infection might be more prominently influenced viral structural proteins, especially the differences in core proteins, rather than host factors.

### Ex vivo Exposure with Different Genotypes of HCV Strains Induced Genotype-specific Decrease of NKG2D Expression on CD56+CD3+ Lymphocytes

**Fig 4:**
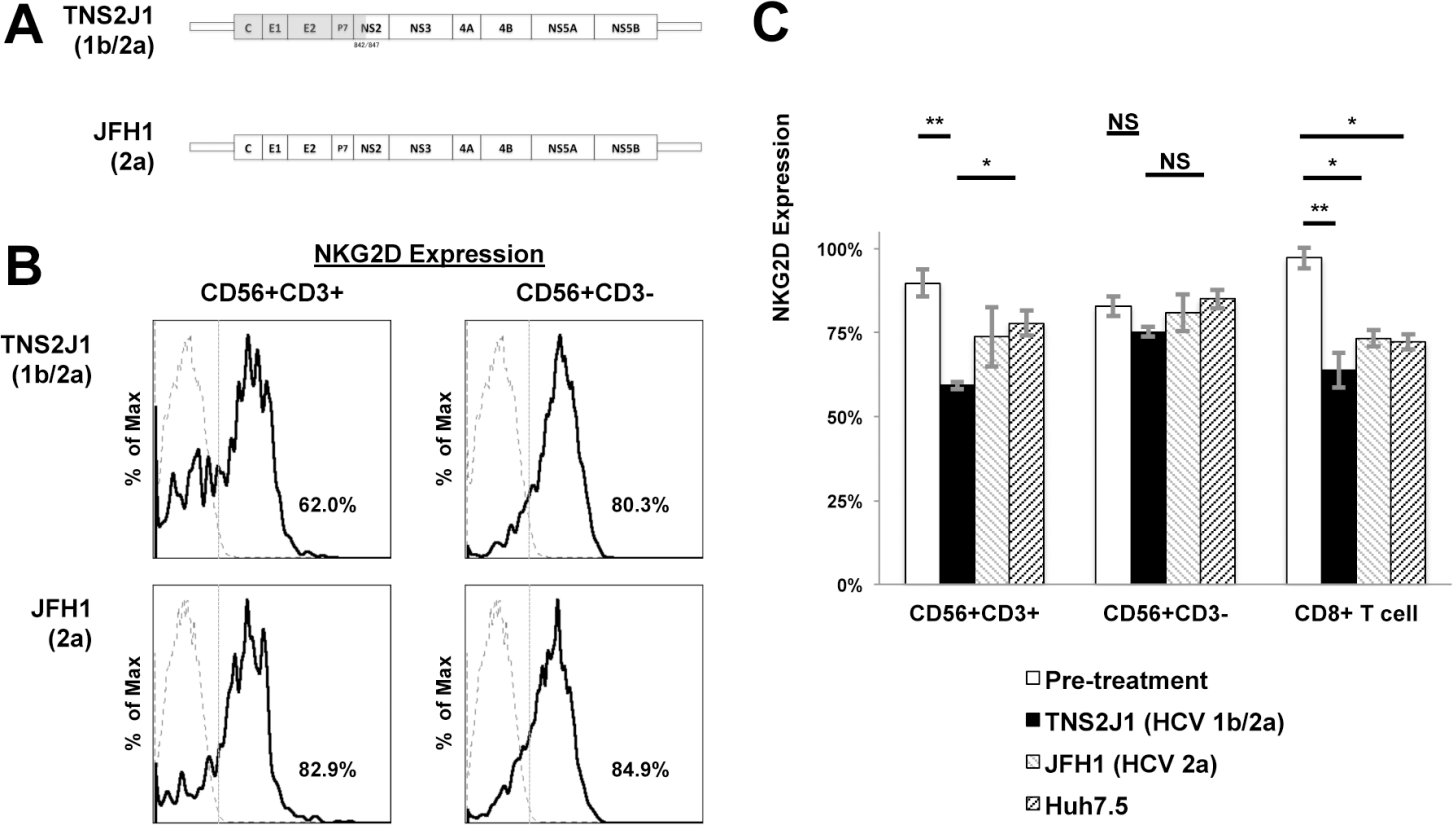
Ex vivo exposure with different genotypes of HCV strains induced genotype-specific decrease of NKG2D expression on CD56+CD3+ lymphocytes. PBMCs from healthy donors were isolated and co-incubated with HCV strains of different genotypes, or with supernatants from Huh7.5 cells for 48 hours, and were analyzed by flow cytometry for NKG2D expression. (A) Genetic schemes of two strains of HCV, TNS2J1, a genotype 1b/2a chimera virus strain, and JFH1, a genotype 2a strain. (B) Representative histograms from THS2J1 (upper) and JFH1 groups (lower) show NKG2D expression on CD56+CD3+ lymphocytes and NK cells. Isotype controls are shown as dashed lines. Percentages show positive NKG2D levels in corresponding compartments. (C) NKG2D expression on CD56+CD3+ lymphocytes, NK or CD8+ CTLs, and representative results of PBMCs from three healthy donors are shown. Data show mean ± SD. *P < 0.05, **P < 0.01, NS: not significant.

As previously described, different genotypes of HCV may influence NKG2D expression on CD56+CD3+ lymphocytes, and may related to structural proteins. Here we used two different strains of HCV genotypes, JHF1 (genotype 2a) and TNS2J1, a chimeric strain of genotype 1b in structural areas and 2a in non-structural areas (Fig 4A), and co-incubate them in vitro with PBMCs from healthy donors. We used whole isolated PBMCs because there is sufficient evidence that immune cells showed interactions between antigen-presenting cells such as dendritic cells are important in sensing viral particles [21]. Using Huh7.5 hepatoma cell line as the control, NKG2D expression on CD56+CD3+ lymphocytes was significantly decreased in the TNS2J1 group, but not in the JFH1 group. This phenomenon was not observed on NK cells (Fig 4B and 4C). In CD8 T lymphocytes, NKG2D expression decreased when co-incubated with both HCV strains; however, this was not statistically significant compared with the group co-incubated with Huh7.5 supernatants (Fig 4C).

### NKG2D Expression on CD56+CD3+ Lymphocytes Predict Treatment Responses to PEG-IFN/Ribavirin Therapy

**Fig 5:**
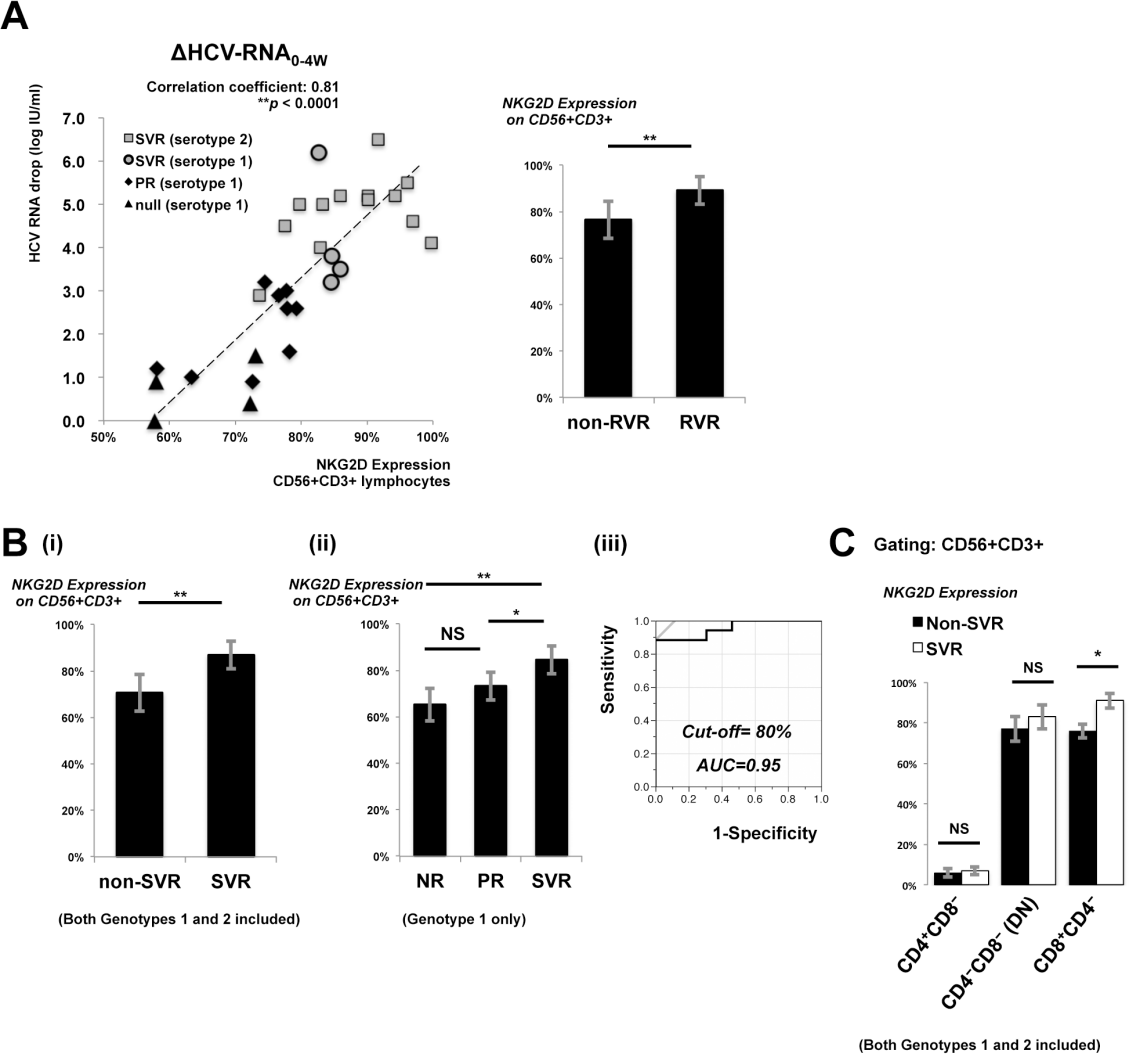
NKG2D expression on CD56+CD3+ lymphocytes predicts treatment responses to PEG-IFN/ ribavirin therapy. PBMCs were collected and analyzed by flow cytometry for NKG2D expression on CD56+CD3+ lymphocytes before treatment. Plasma HCV-RNA levels were measured regularly to monitor viral kinetics during treatment. (A, left) Correlation of decreased plasma HCV RNA (IU/ml) from week 0 to week 4 and NKG2D expression on CD56+CD3+ lymphocytes is shown. (A, right) NKG2D expression was analyzed to compare RVR (n = 8) and non-RVR (n = 22) cases. (B) NKG2D expression on CD56+CD3+ lymphocytes was analyzed to (i) compare SVR (n = 17) and non-SVR (n = 13) cases; (ii) SVR (n = 4), PR (n = 9), and NR (n = 4) in genotype 1 infection; (iii) ROC analysis to predict SVR by NKG2D expression on CD56+CD3+ lymphocytes. (C) Pre-treatment peripheral CD56+CD3+ lymphocytes were stratified into CD4+CD8-, CD8+CD4- and CD4-CD8- double negative (DN) subpopulations. NKG2D expression on corresponding subpopulations were determined. Statistics of NKG2D expression were compared between SVR (n = 17) and non-SVR (n = 13) cases, including both genotypes. Data show mean ± SD. *P < 0.05, **P < 0.01, NS: not significant.

Since HCV genotype 1 infection is more difficult to eradicate with interferon-based therapies than genotype 2, we further investigated whether CD56+CD3+ lymphocytes bearing different NKG2D expressing levels influenced the treatment effect of PEG-IFN/ ribavirin therapy. Thirty patients, including 17 with genotype 1 HCV infection and 13 with genotype 2, were analyzed. PBMCs were isolated and analyzed for NKG2D expression prior to the start of treatment. Regardless of genotype differences, NKG2D expression on CD56+CD3+ lymphocytes was highly and significantly correlated to the degree of HCV-RNA reduction from week 0 to week 4 (ΔHCV-RNA0-4w; correlation coefficient, 0.81; P < 0.0001, Fig 5A, left). When stratified by RVR or not, cases that developed RVR showed significantly higher NKG2D expression on CD56+CD3+ lymphocytes than non-RVR cases (Fig 5A, right).

Significant differences of NKG2D expression on CD56+CD3+ lymphocytes between SVR and non-SVR cases were also observed (Fig 5B-i). When limited to cases of genotype 1, NKG2D expression on CD56+CD3+ lymphocytes from SVR cases was significantly higher than PR or NR (Fig 5B-ii). ROC analysis revealed that NKG2D expression on CD56+CD3+ lymphocytes above 0.80 could predict SVR by PEG-IFN/ ribavirin therapy with a high probability (area under the curve 0.95; Fig 5B-iii). Because NKG2D expression on CD8+ T cells also showed significant difference between HCV genotypes as showed in Fig 1D, we did ROC analysis of NKG2D expression on CD8+ T cells for the prediction of SVR in the same cohort of cases. The area under curve was 0.76 (ROC curve not shown), and was inferior to that of NKG2D expression on CD56+CD3+ lymphocytes.

The pre-treatment NKG2D expression of peripheral CD56+CD3+ lymphocyte subpopulations, ie, CD8+CD4-, CD4+CD8-, or CD4-CD8- double negative (DN) was also analyzed. Higher pre-treatment NKG2D expression of CD8+CD4- subpopulation significantly associated to SVR (Fig 5C).

### Impaired NKG2D Expression on CD56+CD3+ Lymphocytes Correlates to Decreased IFN-γ Production

**Fig 6:**
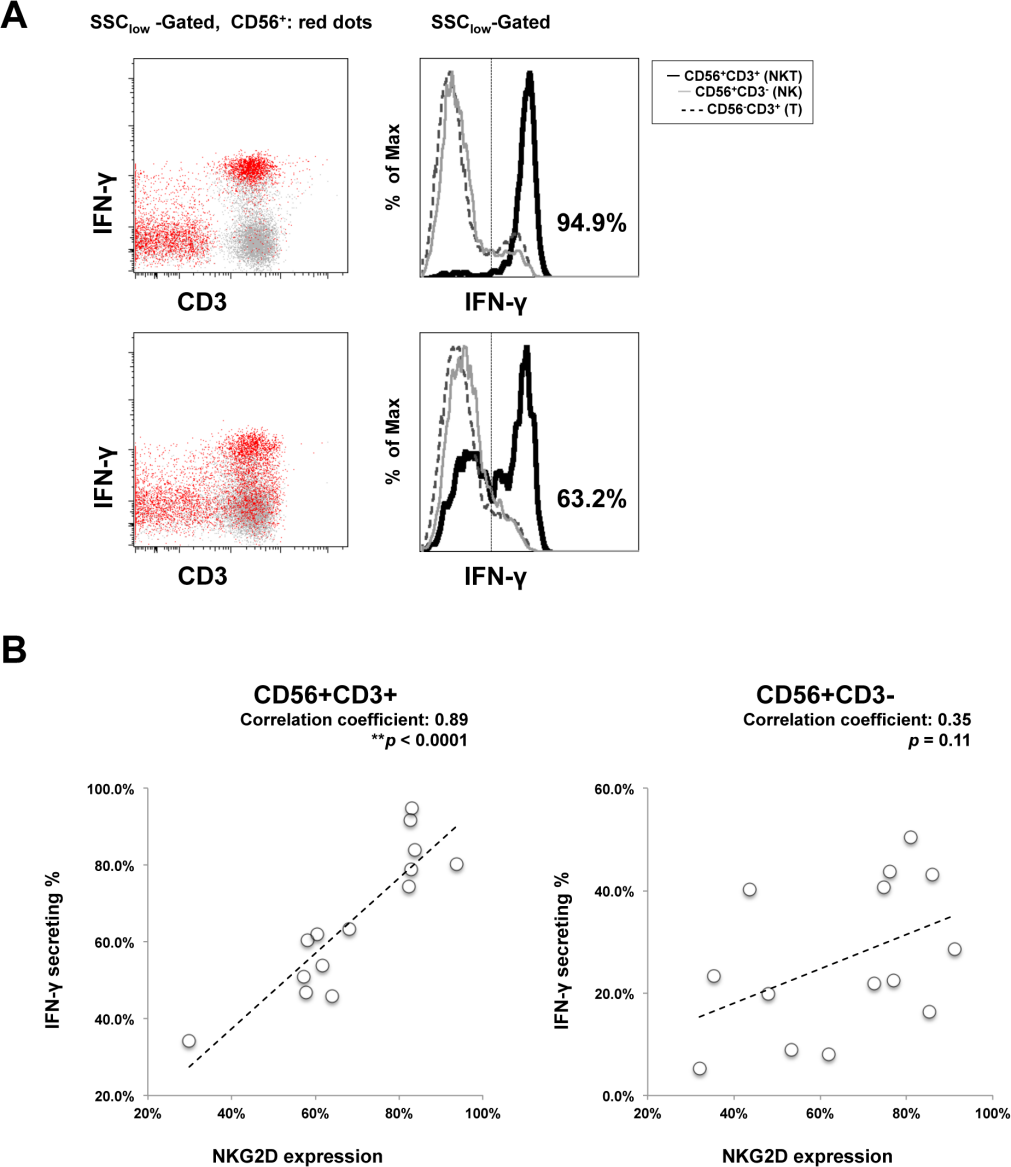
Impaired NKG2D expression on CD56+CD3+ lymphocytes correlates to decreased IFN-γ production. PBMC were collected and analyzed for surface NKG2D expression by flow cytometry in fourteen chronically HCV genotype 1 infected cases. With the same blood sample and at the same time, 4 hours of PMA/ionomycin stimulations were introduced, and then intracellular IFN-γ production levels were determined in CD56+CD3+ (NKT), CD56+CD3- (NK) and CD56-CD3+ (T lymphocyte) populations. (A) Two representative FACS plots and histograms are from the case of SVR (upper) and non-SVR (lower). CD56+ cells are shown as red dots, and other cells as gray dots. n = 14. Statistical correlation of corresponding NKG2D expression and IFN-i production was analyzed in (B).

IFN-γ-mediated killing of HCV-infected hepatocytes is a mechanism of immune-mediated eradication of HCV infection [2, 37], and NKG2D is the receptor for the MICA/MICB pathway that induces IFN-γ production [9]. Therefore, differences of NKG2D expression on CD56+CD3+ lymphocytes that exist between genotypes and within genotype 1 might explain the inferior immunological response. In previous studies, CD56+CD3+ lymphocytes produce more IFN-γ upon activation, compared to NK cells [14] and CD56 negative T cells [37, 38]. To investigate whether differential NKG2D expression within genotype 1 infections on CD56+CD3+ lymphocytes influences IFN-γ production, we analyzed IFN-γ production from CD56+CD3+ lymphocytes after stimulation by PMA/ionomycin (gating strategy in Fig 6A), a known strong stimulator of human peripheral blood CD56+CD3+ lymphocytes [39]. Compared to NK cells, the frequency of IFN-γ- producing CD56+CD3+ lymphocytes was higher within population. Of 14 cases of genotype 1 infection enrolled in this experiment, higher NKG2D expression on CD56+CD3+ lymphocytes significantly correlated with higher IFN-γ production (Fig 6B; correlation coefficient, 0.89; P < 0.0001). However, NKG2D expression on CD56+CD3- NK cells showed no significant correlation with IFN-γ production.

## Discussion

The current study demonstrates that NKG2D expression on CD56+CD3+ lymphocytes, rather than on CD56+CD3− NK cells, is significantly reduced in chronic HCV genotype 1 infection. NKG2D expression on CD56+CD3+ lymphocytes correlates significantly with HCV viral kinetics in the early phase of PEG-IFN/ ribavirin therapy, and predicts treatment responses.

Intrahepatic NKT cells, defined by lymphocytes having both T cell and NK cell surface markers, from HCV-infected individuals showed dysregulated expression of killer immunoglobulin-like receptors [40]. Golden-Mason at al. indicated that inhibitory NKG2A is upregulated in CD56+CD3+ lymphocytes in acute HCV infection, and showed a positive correlation of inhibitory NKG2A expression on CD56+CD3+ lymphocytes to HCV viral load from month 0 and month 6 after infection [41]. Werner et al. showed increased NKG2D expression on peripheral CD1d-restricted NKT cells as part of the innate immune response to acute HCV exposure in healthcare workers who did not develop acute hepatitis [42]. Our current study reveals a not-yet-told correlation of genotype-associated chronicity of HCV persistence with NKG2D expression on CD56+CD3+ lymphocytes. This is the first to specify a single group of immune cells influenced by HCV genotype difference, and may provide an explanation to the natural history of HCV infection.

We also emphasized that the CD56+CD3+ lymphocyte cell subpopulation may also influence treatment response. CD8+ NKT cells exist in humans but are rare in mice [43]. In a previous study, Lin et al. revealed that human peripheral blood CD8+CD56+CD3+ lymphocytes from healthy donors may up-regulate cytotoxic-related surface antigens such as CD16 and NKG2D upon stimulation [44]. Kuylenstierna et al. showed that human peripheral NKT cells express NKG2D almost exclusively on the CD4 negative counterpart, and NKG2D-expressing CD4 negative NKT cells can be activated through NKG2D in a CD1d-independent fashion [11]. On the other hand, it has also been noticed that conventional CD8+ T cells with variant TCRs express NK cell markers upon activation, especially during chronic viral infections such as HIV or CMV [12]. Altogether, though the origin of NKG2D-expessing CD8+CD56+CD3+ lymphocytes might of some dispute, their crucial role in chronic HCV infection is evident for the first time by the current study.

Regulation of NKG2D expression might be multifactorial and both influenced by the virus and the host. HCV core amino acid mutation at aa70 or aa91, a non-double wild type, is known to be predictive to treatment failure in genotype 1 infection [45]. In this study, NKG2D expression on CD56+CD3+ lymphocytes is significantly decreased in non-double wild type cases (Fig 3). We hypothesized that the HCV with different structure proteins may play roles in reduction of NKG2D expression, and this hypothesis was supported by the result of co-incubation of different HCV strains (Fig 4). The co-incubation design has limitations that both direct interaction and humeral factors contained in the supernatants could be possible. HCV infection has been reported to inhibit NK cell function through interaction with CD81 [46, 47] or by the imbalance of cytokines [21]. On the other hand, although we emphasized the genotype-associated difference in NKG2D expression, we also noticed a differential expression of NKG2D within HCV genotype 1 (Fig 1D). Our result didn’t support significant correlation with *IL28B* polymorphisms, however, we cannot rule out the possibility of correlation to other host factors, such as *IFNL4* polymorphisms, which has been reported by Jouvin-Marche et al. about the correlation of favorable genotype with higher early HCV viral load reduction during PEG-IFN/ ribavirin treatment [48]. Recently, Fernandez-Sanchez et al. showed epigenetic modification of histone acetylation determines NKG2D gene expression in NK cells and CTLs in vitro [49], and Quan et al. revealed that HCV core protein may influence this process [50]. Sato et al. showed that inhibition of histone deacetylase suppresses HCV replication [51]. López-Rodríguez et al. found that histone deacetylase polymorphism helps predict IFN-based HCV treatment response [52]. Thus, epigenetic modification of NKG2D expression by HCV proteins may be one possible mechanism. Whether these viral or host factors work on CD56+CD3+ lymphocytes is still unknown, and needs to be further studied.

Type I interferon response are impaired in chronic HCV infection [2, 4], however, exogenous administration of interferon-α during treatment may help eradicate HCV through enforcement of CD56+CD3+ lymphocyte function. Though we observed changes of NKG2D expression in CD56+CD3+ lymphocytes during and after PEG-IFN/ ribavirin treatment (unpublished data), the mechanism how exogenous interferon-α work on NKG2D expression still needs further investigation.

In conclusion, Decreased NKG2D expression on CD56+CD3+ lymphocytes in chronic HCV genotype 1 infection predicts inferior treatment response to PEG-IFN/ribavirin therapy compared to genotype 2. Treatment of chronic hepatitis C progresses rapidly, and IFN-free therapies are becoming new standards in clinical settings. However, analysis of NKG2D expression on peripheral CD56+CD3+ lymphocytes may provide better stratification of treatable patients with genotype 1 infection who may not be suitable for direct-antiviral agents. Furthermore, the better understanding of the mechanisms how HCV interacts with host immunity may provide some hints for possible treatment to HCV infections and its related complications.

## Supporting Information

S1 Fig General gating strategy. (TIF)

S1 Table Detailed clinical characteristics of cases recruited in the cohort of NKG2D expression evaluation. (DOCX)

S2 Table Detailed clinical characteristics of cases recruited in the cohort of treatment response evaluation. (DOCX)

S3 Table Clinical characteristics of cases recruited in the cohort of treatment response evaluation, stratified with SVR and non-SVR. (DOCX)

S4 Table Clinical characteristics of cases recruited in the cohort of treatment response evaluation (genotype 1 only), stratified with NR, PR, and SVR. (DOCX)

S5 Table Clinical characteristics of cases for ex vivo IFN-γ secretion evaluation. (DOCX)
