# Supplementary figures and images for: Genotype-Associated Differential NKG2D Expression on CD56+CD3+ Lymphocytes Predicts Response to Pegylated-Interferon/ Ribavirin Therapy in Chronic Hepatitis C
Source: PLoS One. 2015 May 12;10(5):e0125664. doi: 10.1371/journal.pone.0125664 (PMC4428701; doi:10.1371/journal.pone.0125664)

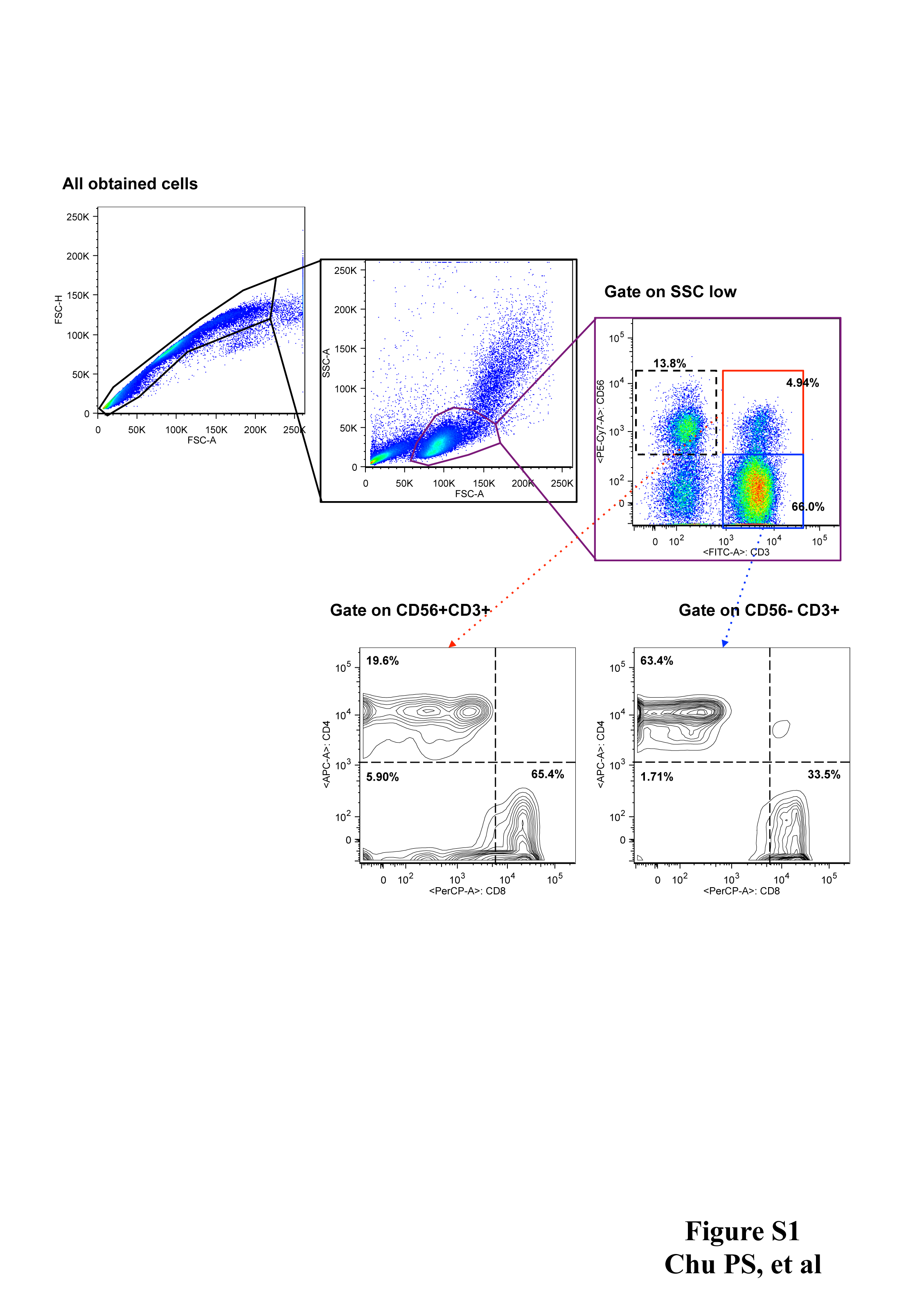

Supplement: S1 Fig — (TIF) [file pone.0125664.s001.tif]
