# Supplementary material for: Genotype-Associated Differential NKG2D Expression on CD56+CD3+ Lymphocytes Predicts Response to Pegylated-Interferon/ Ribavirin Therapy in Chronic Hepatitis C
Source: PLoS One. 2015 May 12;10(5):e0125664. doi: 10.1371/journal.pone.0125664 (PMC4428701; doi:10.1371/journal.pone.0125664)
Supplement: S1 Table — (DOCX) [file pone.0125664.s002.docx]

Supporting Materials- 1

**Table S1. Detailed clinical characteristics of cases recruited in the cohort of NKG2D expression evaluation.**

| Number | Gender | Age | HCV genotype | HCV-RNA | T-Bil | Albumin | WBC | ALT | ALP | GGTP | Platelet | type 4 COL7s | HbA1c | T-CHO | NKG2D exp CD56+CD3+ | NKG2D exp CD56+CD3- | NKG2D exp CD8+ T cell |
| --- | --- | --- | --- | --- | --- | --- | --- | --- | --- | --- | --- | --- | --- | --- | --- | --- | --- |
| 1 | 2 | 69 | 1 | 7.2 | 0.8 | 4.3 | 6000 | 41 | 267 | 18 | 145 | NA | NA | 161 | 0.766 | 0.902 | 0.947 |
| 2 | 2 | 55 | 1 | 6.1 | 0.6 | 3.6 | 3000 | 121 | 534 | 87 | 84 | 9.1 | 6.8 | 150 | 0.634 | 0.325 | 0.732 |
| 3 | 2 | 52 | 1 | 6.5 | 0.6 | 3.8 | 3700 | 29 | 272 | 17 | 167 | 6.2 | 4.9 | 149 | 0.681 | 0.406 | 0.841 |
| 4 | 2 | 61 | 2 | 6.7 | 0.8 | 3.9 | 6900 | 25 | 346 | 44 | 244 | 4.3 | 6.1 | 212 | 0.997 | 0.493 | 0.999 |
| 5 | 2 | 63 | 2 | 6.4 | 0.7 | 4.2 | 4500 | 11 | 227 | 13 | 205 | 4.1 | 4.1 | 185 | 0.943 | 0.536 | 0.929 |
| 6 | 2 | 62 | 1 | 6.7 | 0.8 | 4.3 | 3300 | 91 | 363 | 102 | 81 | 9.1 | 5.1 | 157 | 0.723 | 0.391 | 0.749 |
| 7 | 2 | 67 | 2 | 6.7 | 0.7 | 4.3 | 3800 | 54 | 272 | 28 | 104 | 5.7 | 4.9 | 166 | 0.901 | 0.501 | 0.904 |
| 8 | 1 | 61 | 2 | 4.5 | 1 | 4.1 | 4100 | 23 | 210 | 31 | 174 | 5 | 5.5 | 162 | 0.775 | 0.687 | 0.905 |
| 9 | 2 | 58 | 1 | 7.7 | 1.3 | 4 | 2000 | 69 | 211 | 26 | 80 | 6.8 | 5 | 139 | 0.731 | 0.399 | 0.695 |
| 10 | 1 | 45 | 2 | 5.5 | 0.6 | 4.4 | 7100 | 60 | 612 | 127 | 164 | 5 | 5.4 | 175 | 0.97 | 0.445 | 0.939 |
| 11 | 2 | 48 | 2 | 6.5 | 1.1 | 4.1 | 6500 | 23 | 209 | 22 | 165 | 4.3 | 5 | 156 | 0.961 | 0.795 | 0.946 |
| 12 | 2 | 44 | 2 | 6.5 | 0.7 | 3.8 | 3500 | 192 | 331 | 44 | 206 | NA | 4.8 | 119 | 0.901 | 0.73 | 0.905 |
| 13 | 1 | 65 | 1 | 7.2 | 0.7 | 4.6 | 8200 | 32 | 275 | 30 | 190 | 3.8 | NA | 181 | 0.696 | 0.321 | 0.921 |
| 14 | 2 | 51 | 1 | 6.6 | 0.6 | 4.1 | 3900 | 29 | 278 | 55 | 177 | 4.8 | 5.7 | 176 | 0.486 | 0.595 | 0.922 |
| 15 | 2 | 25 | 2 | 6.5 | 0.6 | 4.3 | 5700 | 189 | 195 | 71 | 263 | 4.3 | 4.7 | 175 | 0.917 | 0.873 | 0.982 |
| 16 | 2 | 62 | 1 | 6.3 | 0.8 | 3.2 | 5400 | 38 | 165 | 21 | 140 | 6.1 | 5 | 152 | 0.58 | 0.69 | 0.862 |
| 17 | 1 | 45 | 1 | 5.4 | 0.9 | 3.6 | 8500 | 91 | 262 | 37 | 231 | 6.8 | 5.4 | 119 | 0.726 | 0.713 | 0.956 |
| 18 | 2 | 36 | 1 | 6 | 0.9 | 4.8 | 5300 | 50 | 195 | 18 | 236 | 4.1 | 4.9 | 191 | 0.805 | 0.791 | 0.93 |
| 19 | 2 | 77 | 1 | 6.8 | 1.4 | 3.8 | 2400 | 35 | 159 | 33 | 105 | 6 | 5.3 | 123 | 0.778 | 0.856 | 0.942 |
| 20 | 1 | 65 | 1 | 7 | 1.7 | 4.2 | 6000 | 66 | 230 | 59 | 224 | 5.5 | 6.1 | 252 | 0.538 | 0.327 | 0.56 |
| 21 | 1 | 72 | 1 | 6.3 | 0.8 | 3.4 | 4000 | 179 | 201 | 49 | 157 | 7.4 | 5.1 | 156 | 0.859 | 0.916 | 0.934 |
| 22 | 2 | 52 | 1 | 5.7 | 1.3 | 4.3 | 3900 | 61 | 280 | 67 | 91 | 8 | 5.7 | 221 | 0.779 | 0.665 | 0.833 |
| 23 | 1 | 79 | 1 | 6 | 1.5 | 3.3 | 2200 | 37 | 272 | 107 | 38 | 11 | NA | 113 | 0.926 | 0.946 | 0.942 |
| 24 | 2 | 67 | 1 | 7.4 | 1.7 | 4.1 | 4000 | 20 | 468 | 22 | 296 | 4.6 | NA | 140 | 0.827 | 0.838 | 0.843 |
| 25 | 1 | 75 | 1 | 6.8 | 0.8 | 3.4 | 5400 | 100 | 603 | 66 | 143 | 13 | 4.3 | 131 | 0.821 | 0.804 | 0.947 |
| 26 | 2 | 54 | 1 | 7 | 0.5 | 4.2 | 4500 | 35 | 349 | 46 | 143 | NA | 6.8 | 172 | 0.826 | 0.732 | 0.919 |
| 27 | 1 | 59 | 2 | 7 | 0.6 | 4.5 | 4600 | 22 | 196 | 16 | 233 | 4.5 | 4.9 | 193 | 0.829 | 0.809 | 0.968 |
| 28 | 2 | 71 | 1 | 6 | 0.7 | 4.4 | 5300 | 22 | 200 | 19 | 238 | 4.2 | 5.3 | 214 | 0.927 | 0.747 | 0.972 |
| 29 | 1 | 65 | 1 | 4.3 | 0.8 | 4.1 | 3900 | 75 | 203 | 73 | 212 | 5.2 | 4.6 | 157 | 0.616 | 0.354 | 0.734 |
| 30 | 1 | 32 | 1 | 5.4 | 1 | 4.6 | 3900 | 96 | 222 | 31 | 152 | 2.8 | 5.1 | 191 | 0.83 | 0.852 | 0.953 |
| 31 | 1 | 57 | 1 | 7.3 | 0.8 | 4.3 | 6100 | 54 | 187 | 24 | 197 | 4.2 | NA | 179 | 0.823 | 0.761 | 0.971 |
| 32 | 1 | 47 | 1 | 7.4 | 0.7 | 4.8 | 5900 | 38 | 159 | 74 | 162 | NA | 7 | 140 | 0.681 | 0.725 | 0.838 |
| 33 | 1 | 53 | 1 | 5.6 | 0.7 | 3.5 | 6100 | 75 | 232 | 180 | 165 | NA | 5.7 | 191 | 0.581 | 0.48 | 0.719 |
| 34 | 1 | 59 | 1 | 6.8 | 0.8 | 3.4 | 3400 | 67 | 222 | 103 | 82 | 7.9 | 4.8 | 143 | 0.299 | 0.32 | 0.35 |
| 35 | 1 | 68 | 1 | 6 | 0.6 | 3.8 | 3000 | 33 | 302 | 13 | 107 | 6.4 | 4.9 | 144 | 0.693 | 0.485 | 0.701 |
| 36 | 1 | 58 | 1 | 7.3 | 0.7 | 3.9 | 7400 | 22 | 127 | 31 | 246 | 3.8 | 5.5 | 248 | 0.759 | 0.554 | 0.89 |
| 37 | 2 | 64 | 1 | 6.7 | 0.9 | 4.1 | 5200 | 34 | 386 | 21 | 134 | 5.6 | 5.2 | 158 | 0.745 | 0.58 | 0.757 |
| 38 | 2 | 69 | 1 | 5.2 | 0.8 | 4 | 2800 | 43 | 205 | 17 | 187 | 3.2 | 5.4 | 215 | 0.845 | 0.924 | 0.696 |
| 39 | 1 | 48 | 1 | 7 | 1.1 | 4 | 3600 | 18 | 174 | 21 | 170 | 2.7 | 5 | 186 | 0.554 | 0.49 | 0.658 |
| 40 | 1 | 48 | 2 | 6.3 | 2.3 | 3.4 | 2400 | 214 | 529 | 61 | 66 | 10 | 4.6 | 136 | 0.737 | 0.82 | 0.868 |
| 41 | 1 | 62 | 1 | 6.6 | 1 | 3.8 | 6000 | 49 | 179 | 34 | 167 | 4.7 | 5.6 | 230 | 0.848 | 0.856 | 0.928 |
| 42 | 1 | 76 | 2 | 6.6 | 0.6 | 4.1 | 4200 | 45 | 293 | 36 | 168 | 3.6 | 5.5 | 146 | 0.859 | 0.818 | 0.924 |
| 43 | 1 | 77 | 1 |  | 1 | 2.7 | 4500 | 87 | 510 | 86 | 100 | 13 | 7.1 | 140 | 0.573 | 0.437 | 0.565 |
| 44 | 1 | 68 | 1 | 7.4 | 0.8 | 4.4 | 5300 | 23 | 223 | 28 | 203 | NA | NA | 150 | 0.578 | 0.534 | 0.871 |
| 45 | 1 | 34 | 1 | 7.1 | 0.7 | 4.5 | 4600 | 28 | 220 | 102 | 210 | 4 | 5.7 | 164 | 0.838 | 0.912 | 0.933 |
| 46 | 2 | 67 | 2 | 6.2 | 0.6 | 3.6 | 3500 | 113 | 156 | 26 | 230 | NA | 5.1 | 176 | 0.797 | 0.77 | 0.9 |
| 47 | 2 | 45 | 1 | 7.5 | 2 | 4.4 | 6400 | 49 | 183 | 35 | 177 | NA | 4.4 | 233 | 0.828 | 0.86 | 0.985 |
| 48 | 2 | 59 | 2 | 6.4 | 0.5 | 3.8 | 3300 | 17 | 240 | 16 | 229 | 3.7 | 5.2 | 221 | 0.832 | 0.796 | 0.938 |
| 49 | 2 | 47 | 1 | 6.7 | 0.8 | 4.6 | 5100 | 27 | 215 | 16 | 247 | NA | NA | 250 | 0.885 | 0.869 | 0.959 |
| 50 | 1 | 63 | 1 | 7 | 2.6 | 3.8 | 3000 | 24 | 106 | 30 | 183 | 4 | 5.1 | 194 | 0.756 | 0.574 | 0.877 |
| 51 | 1 | 68 | 1 | 6.7 | 0.6 | 4.2 | 3700 | 46 | 194 | 60 | 164 | NA | NA | 181 | 0.749 | 0.511 | 0.789 |
| 52 | 1 | 65 | 1 | 5.9 | 0.9 | 3.9 | 7600 | 24 | 319 | 102 | 232 | NA | 5.4 | 169 | 0.782 | 0.502 | 0.862 |
| 53 | 1 | 57 | 1 | 7.5 | 1.1 | 4.8 | 6400 | 23 | 176 | 31 | 241 | 3.5 | 6.9 | 206 | 0.623 | 0.669 | 0.825 |
| 54 | 1 | 58 | 1 | 6.7 | 1 | 3.3 | 2700 | 168 | 337 | 63 | 106 | 11 | 5.1 | 152 | 0.647 | 0.893 | 0.776 |
| 55 | 2 | 62 | 1 | 6.5 | 1 | 4.4 | 4700 | 10 | 216 | 19 | 203 | 4.1 | 5.1 | 272 | 0.478 | 0.534 | 0.692 |
| 56 | 2 | 77 | 1 | 6.9 | 0.7 | 4.1 | 5200 | 37 | 283 | 36 | 133 | NA | 5.5 | 176 | 0.793 | 0.598 | 0.858 |
| 57 | 2 | 60 | 1 | 6.6 | 0.6 | 3.3 | 4600 | 266 | 397 | 35 | 151 | 7.7 | 5.3 | 133 | 0.686 | 0.596 | 0.615 |
| 58 | 1 | 69 | 1 | 6.9 | 1.6 | 4.3 | 5200 | 19 | 190 | 21 | 191 | 4.8 | 5.3 | 174 | 0.604 | 0.442 | 0.722 |
| 59 | 1 | 62 | 1 | 5.7 | 0.7 | 3.6 | 4100 | 31 | 23 | 83 | 214 | 3.6 | 5 | 175 | 0.678 | 0.591 | 0.797 |
| 60 | 2 | 70 | 1 | 6.6 | 0.8 | 4.4 | 4700 | 44 | 250 | 21 | 141 | NA | NA | 203 | 0.638 | 0.799 | 0.82 |
| 61 | 2 | 47 | 1 | 6.1 | 2.7 | 3.6 | 5300 | 77 | 223 | 66 | 116 | 10 | 4.9 | 134 | 0.572 | 0.711 | 0.759 |
| 62 | 1 | 63 | 1 | 6.4 | 1 | 4.4 | 4800 | 308 | 285 | 372 | 144 | 8.1 | 4.9 | 204 | 0.645 | 0.789 | 0.868 |
| 63 | 1 | 48 | 1 | 7.1 | 0.5 | 4.4 | 2800 | 32 | 29 | 121 | 93 | 7.4 | 4.9 | 130 | 0.631 | 0.635 | 0.815 |
| 64 | 2 | 50 | 1 | 3.8 | 0.9 | 4.2 | 6200 | 82 | 262 | 25 | 185 | 4.2 | 5.3 | 240 | 0.847 | 0.836 | 0.966 |
| 65 | 1 | 69 | 1 | 6.6 | 0.5 | 3.9 | 5000 | 40 | 179 | 27 | 184 | 3.3 | 5.4 | 187 | 0.71 | 0.625 | 0.93 |
| 66 | 1 | 59 | 1 | 6.9 | 0.6 | 4.2 | 4700 | 40 | 133 | 55 | 145 | 5 | 5.8 | 177 | 0.68 | 0.74 | 0.84 |

Abbreviations: ALT, alanine aminotransferase, COL, collagen, Bil, bilirubin, GTP，glutamine transpeptidase, CHO, cholesterol, NA, not available.
