# Supplementary material for: Genotype-Associated Differential NKG2D Expression on CD56+CD3+ Lymphocytes Predicts Response to Pegylated-Interferon/ Ribavirin Therapy in Chronic Hepatitis C
Source: PLoS One. 2015 May 12;10(5):e0125664. doi: 10.1371/journal.pone.0125664 (PMC4428701; doi:10.1371/journal.pone.0125664)
Supplement: S2 Table — (DOCX) [file pone.0125664.s003.docx]

**Table S2. Detailed clinical characteristics of cases recruited in the cohort of treatment response evaluation.**

| Case No. | Gender | Age | Genotype | RVR | SVR | HCV-RNA | Fibrosis Stage | ALT | Albumin | PLT | 4COL7s | GGTP | T-Bil | HBA1c | T-CHO | WBC |
| --- | --- | --- | --- | --- | --- | --- | --- | --- | --- | --- | --- | --- | --- | --- | --- | --- |
| 1 | F | 69 | 1 | no | PR | 7.2 | NA | 41 | 4.3 | 145 | 6.7 | 18 | 0.8 | 5.6 | 161 | 6000 |
| 2 | F | 55 | 1 | no | PR | 6.1 | F2 | 121 | 3.6 | 84 | 9.1 | 87 | 0.6 | 7.2 | 150 | 3000 |
| 3 | F | 61 | 2 | no | SVR | 6.7 | F2 | 25 | 3.9 | 244 | 4.3 | 44 | 0.8 | 6.5 | 212 | 6900 |
| 4 | F | 63 | 2 | yes | SVR | 6.4 | F1 | 11 | 4.2 | 205 | 4.1 | 13 | 0.7 | 4.5 | 185 | 4500 |
| 5 | F | 62 | 1 | no | NR | 6.7 | F4 | 91 | 4.3 | 81 | 9.1 | 102 | 0.8 | 5.5 | 157 | 3300 |
| 6 | F | 67 | 2 | no | SVR | 6.7 | F3 | 54 | 4.3 | 104 | 5.7 | 28 | 0.7 | 5.3 | 166 | 3800 |
| 7 | M | 61 | 2 | yes | SVR | 4.5 | F2 | 23 | 4.1 | 174 | 5.0 | 31 | 1.0 | 5.9 | 162 | 4100 |
| 8 | F | 58 | 1 | no | NR | 7.7 | F3 | 69 | 4.0 | 80 | 6.8 | 26 | 1.3 | 5.4 | 139 | 2000 |
| 9 | F | 45 | 2 | yes | SVR | 5.5 | F2 | 60 | 4.4 | 164 | 5.0 | 127 | 0.6 | 5.8 | 175 | 7100 |
| 10 | F | 48 | 2 | yes | SVR | 6.5 | F1 | 23 | 4.1 | 165 | 4.3 | 22 | 1.1 | 5.4 | 156 | 6500 |
| 11 | F | 44 | 2 | yes | SVR | 6.5 | NA | 192 | 3.8 | 206 | 5.7 | 44 | 0.7 | 5.2 | 119 | 3500 |
| 12 | F | 26 | 2 | yes | SVR | 6.5 | NA | 189 | 4.3 | 263 | 4.3 | 71 | 0.6 | 5.1 | 175 | 5700 |
| 13 | F | 62 | 1 | no | NR | 6.3 | F3 | 38 | 3.2 | 140 | 6.1 | 21 | 0.8 | 5.4 | 152 | 5400 |
| 14 | M | 45 | 1 | no | PR | 5.4 | F3 | 91 | 3.6 | 231 | 6.8 | 37 | 0.9 | 5.8 | 119 | 8500 |
| 15 | F | 77 | 1 | no | PR | 6.8 | NA | 35 | 3.8 | 105 | 6.0 | 33 | 1.4 | 5.7 | 123 | 2400 |
| 16 | M | 72 | 1 | no | SVR | 6.3 | F2 | 179 | 3.4 | 157 | 7.4 | 49 | 0.8 | 5.5 | 156 | 4000 |
| 17 | F | 52 | 1 | no | PR | 5.7 | F4 | 61 | 4.3 | 91 | 8.0 | 67 | 0.8 | 6.1 | 221 | 3900 |
| 18 | F | 67 | 1 | yes | SVR | 7.4 | NA | 20 | 4.1 | 296 | 4.6 | 22 | 1.7 | 5.6 | 160 | 4000 |
| 19 | M | 59 | 2 | no | SVR | 7.0 | F1 | 22 | 4.5 | 233 | 4.5 | 16 | 0.6 | 5.3 | 193 | 4600 |
| 20 | M | 76 | 2 | no | SVR | 6.6 | F2 | 45 | 4.1 | 168 | 3.6 | 36 | 0.6 | 5.9 | 146 | 4200 |
| 21 | F | 67 | 2 | no | SVR | 6.2 | NA | 113 | 3.6 | 230 | 4.7 | 26 | 0.6 | 5.5 | 176 | 3500 |
| 22 | F | 59 | 2 | no | SVR | 6.4 | NA | 17 | 3.8 | 229 | 3.7 | 16 | 0.5 | 5.6 | 221 | 3300 |
| 23 | M | 53 | 1 | no | PR | 6.7 | NA | 75 | 3.5 | 165 | 5.7 | 180 | 0.7 | 5.7 | 191 | 6100 |
| 24 | F | 64 | 1 | no | PR | 6.8 | F2 | 34 | 4.1 | 134 | 5.6 | 21 | 0.9 | 5.2 | 158 | 5200 |
| 25 | F | 69 | 1 | no | SVR | 5.2 | F3 | 43 | 4.0 | 187 | 3.2 | 17 | 0.9 | 5.2 | 187 | 2800 |
| 26 | M | 48 | 2 | no | SVR | 6.3 | F4 | 214 | 3.4 | 66 | 10 | 61 | 2.3 | 4.6 | 136 | 2400 |
| 27 | M | 68 | 1 | no | NR | 7.4 | NA | 23 | 4.4 | 203 | 5.7 | 28 | 0.8 | 5.6 | 164 | 5300 |
| 28 | M | 65 | 1 | no | PR | 5.9 | NA | 24 | 3.9 | 232 | 5.3 | 102 | 0.9 | 5.4 | 169 | 7600 |
| 29 | F | 77 | 1 | no | PR | 7.0 | NA | 37 | 4.1 | 133 | 6.3 | 37 | 0.7 | 5.5 | 176 | 5200 |
| 30 | F | 50 | 1 | yes | SVR | 3.8 | F1 | 82 | 4.2 | 185 | 4.2 | 25 | 0.9 | 5.3 | 240 | 6200 |

Abbreviations: ALT, alanine aminotransferase, PLT, platelet, COL, collagen, Bil, bilirubin, GTP，glutamine transpeptidase, CHO, cholesterol.
