# Supplementary material for: Genotype-Associated Differential NKG2D Expression on CD56+CD3+ Lymphocytes Predicts Response to Pegylated-Interferon/ Ribavirin Therapy in Chronic Hepatitis C
Source: PLoS One. 2015 May 12;10(5):e0125664. doi: 10.1371/journal.pone.0125664 (PMC4428701; doi:10.1371/journal.pone.0125664)
Supplement: S3 Table — (DOCX) [file pone.0125664.s004.docx]

**Table S3. Clinical characteristics of cases recruited in the cohort of treatment response evaluation, stratified with SVR and non-SVR.**

| **Characteristics** | **Units** | **All cases** |  |  | ***p*** |
| --- | --- | --- | --- | --- | --- |
| **Non-SVR: SVR** | - | 13: 17 | **Non-SVR** | **SVR** | **-** |
| **Patient number, n** |  | 30 | 13 | 17 | **-** |
| **Age** | years | Median 61.5  (51.5-67.25) | Median 62  (54-68.5) | Median 61  (48-67) | **0.31^a^** |
| **Gender, M:F** | - | 10:20 | 4:9 | 6:11 | **0.79^b^** |
| **HCV genotype, 1: 2** | - | 17:13 | 13:0 | 4:13 | **<0.0001**^b^** |
| **Liver Histology**  **F1/F2/F3/F4**  **(data unavailable)** | - | 4/7/5/3 (11) | 0/2/3/2 (6) | 4/5/2/1 (5) | **0.18^b^** |
| **HCV-RNA** | Log IU/ml | Median 6.5  (6.1-6.7) | Median 6.7  (6.0-7.0) | Median 6.4  (5.9-6.7) | **0.16^a^** |
| **PLT count** | x1000/μl | 170±60 | 140±54 | 193±56 | **0.016*^c^** |
| **ALT** | IU/L | 68±58 | 57±31 | 77±72 | **0.35^c^** |

^a^ Statistics are analyzed by Mann-Whitney U-test. IQRs are shown in the parentheses.

^b^ Statistics are analyzed by Fisher’s exact test.

^c^ Statistics are analyzed by Student’s t-test. Data are shown as mean± standard deviation.

Abbreviations: SVR, sustained viral responder, ALT, alanine aminotransferase, ALP, alkaline phosphatase, GTP，glutamine transpeptidase.
