# Supplementary material for: Genotype-Associated Differential NKG2D Expression on CD56+CD3+ Lymphocytes Predicts Response to Pegylated-Interferon/ Ribavirin Therapy in Chronic Hepatitis C
Source: PLoS One. 2015 May 12;10(5):e0125664. doi: 10.1371/journal.pone.0125664 (PMC4428701; doi:10.1371/journal.pone.0125664)
Supplement: S4 Table — (DOCX) [file pone.0125664.s005.docx]

**Table S4. Clinical characteristics of cases recruited in the cohort of treatment response evaluation (genotype 1 only), stratified with NR, PR, and SVR.**

| **Characteristics** | **Units** | **genotype 1** |  |  |  | ***p*** |
| --- | --- | --- | --- | --- | --- | --- |
| **NR: PR: SVR** | - | 4: 9: 4 | **NR** | **PR** | **SVR** | **-** |
| **Patient number, n** |  | 17 | 4 | 9 | 4 | **-** |
| **Age** | years | Median 64  (54-69) | 62.5±5.0 | 61.9±3.3 | 64.5±5.0 | **0.91^a^** |
| **Gender, M:F** | - | 5:12 | 1:3 | 3:6 | 1:3 | **0.93^b^** |
| **HCV-RNA** | Log IU/ml | Median 6.7  (5.8-7.0) | 7.0±0.5 | 6.4±0.3 | 5.7±0.5 | **0.14^a^** |
| **Liver Histology,**  **F1/F2/F3/F4**  **(data unavailable)** | - | 1/3/4/2 (7) | 0/0/2/1 (1) | 0/2/1/1 (5) | 1/1/1/0 (1) | **0.48^b^** |
| **PLT count** | x1000/μl | 156±61 | 126±28 | 147±19 | 206±28 | **0.14^c^** |
| **ALT** | IU/L | 63±42 | 55±21 | 58±14 | 81±22 | **0.63^c^** |

^a^ Statistics are analyzed by Mann-Whitney U-test. IQRs are shown in the parentheses.

^b^ Statistics are analyzed by Fisher’s exact test.

^c^ Statistics are analyzed by Student’s t-test. Data are shown as mean± standard deviation.

Abbreviations: NR, null responder, PR, partial responder, SVR, sustained viral responder, ALT, alanine aminotransferase, ALP, alkaline phosphatase, GTP，glutamine transpeptidase.
