# Supplementary material for: Genotype-Associated Differential NKG2D Expression on CD56+CD3+ Lymphocytes Predicts Response to Pegylated-Interferon/ Ribavirin Therapy in Chronic Hepatitis C
Source: PLoS One. 2015 May 12;10(5):e0125664. doi: 10.1371/journal.pone.0125664 (PMC4428701; doi:10.1371/journal.pone.0125664)
Supplement: S5 Table — (DOCX) [file pone.0125664.s006.docx]

**Table S5. Clinical characteristics of cases for ex vivo IFN-γ secretion evaluation.**

|  | Units |  |
| --- | --- | --- |
| Patient number |  | 14 |
| Age^a^ | years | Median 59 (47-67) |
| Gender, M: F | - | 10:4 |
| T-Bil | mg/dl | 0.86±0.38 |
| HCV-RNA^a^ | Log IU/ml | Median 7.0 (6.0-7.3) |
| Platelet count | x1000/mcl | 181±47 |
| Serum type 4 collagen 7s | IU/L | 5.7±3.3 |
| Serum albumin | g/dl | 4.1±0.6 |
| HbA1c | % | 5.8±0.9 |
| Total cholesterol | mg/dl | 180±29 |
| ALT | IU/L | 57±29 |
| ALP | IU/L | 234±93 |
| γ-GTP | IU/L | 58±47 |
| WBC | /mcl | 4990±1070 |

^a^ Data are shown with median and IQRs in the parentheses.

Other data are shown as mean± SD. Abbreviations: ALT, alanine aminotransferase, ALP, alkaline phosphatase, GTP，glutamine transpeptidase.
